# Using aided cortical assessment as an objective tool to evaluate cochlear implant fitting in users with single-sided deafness

**DOI:** 10.1371/journal.pone.0193081

**Published:** 2018-02-22

**Authors:** Dayse Távora-Vieira, Andre Wedekind, Roberta Marino, Suzanne C. Purdy, Gunesh P. Rajan

**Affiliations:** 1 Otolaryngology, Head & Neck Surgery, School of Surgery, University of Western Australia, Perth, Australia; 2 Fiona Stanley Hospital, Perth, Australia; 3 School of Physhology, Faculty of Science, University of Auckland, Auckland, New Zealand; 4 Eisdell Moore Centre, Hearing and Balance Research, Auckland, New Zealand; The Ohio State University, UNITED STATES

## Abstract

**Objectives:**

To assess the use of cortical auditory evoked potentials (CAEPs) to verify, and if necessary, optimize the cochlear implant (CI) fitting of adult CI users with postlingual single-sided deafness (SSD).

**Methods:**

Sound field cortical responses to the speech tokens /m/, /g/, /t/, and /s/ were recorded from input to the CI while the normal hearing ear was masked. Responses were evaluated by visual inspection and classified as presence or absence of the CAEPs components P1, N1, P2. In case of an absence fitting was adjusted accordingly. After fitting, subjects were asked to use their new setting for 2–3 weeks for acclimatization purposes and then return for retesting. At retesting, new CAEP recordings were performed to objectively ensure that the new fitting maps effectively activated the auditory cortex.

**Results:**

In 14/19 subjects, as per visual inspection, clear CAEPs were recorded by each speech token and were, therefore, not refit. In the other 5 subjects, CAEPs could not be evoked for at least one speech token. The fitting maps in these subjects were adjusted until clear CAEPs were evoked for all 4 speech tokens.

**Conclusions:**

CAEP can be used to quickly and objectively verify the suitability of CI fitting in experienced adult CI users with SSD. If used in the early post-implantation stage, this method could help CI users derive greater benefit for CI use and, therefore, be more committed to auditory training.

## Introduction

Cochlear implantation is the most efficacious treatment for most people with bilateral severe or severe-to-profound deafness [1,2]. The hearing performance of cochlear implant (CI) users, while influenced by several physical and psychosocial factors, has benefitted from improved surgical techniques, electrode array design, and programming strategies [3].

Over time, CI indications have been broadened to include persons with residual hearing and those with “ski-slope” hearing loss who derive limited benefit from conventional amplification, e.g. hearing aids [4–6]. The most recent expansion in candidacy criteria is for people with single-sided deafness (SSD), which is defined as having normal hearing (PTA ≤30 dB HL) in one ear but severe or profound hearing loss (PTA ≥70 dB HL) in the contralateral ear [7]. In several regions such as continental Europe and Australia CI is recognized and approved as a treatment option for SSD while in various countries such as the U.S.A. CI for SSD is still an off-label use of the device.

Since the late 2000s there has been a rapid increase in the number of studies investigating the benefits of CI use in people with SSD with and without tinnitus. There is strong evidence that CI provision is the only treatment option that can provide the benefits of binaural hearing in SSD and treat tinnitus, if present [8–18]. Additionally, compared to other treatment modalities, CIs allow a greater speech understanding in noise and in localization ability [19,20].

However, to the best of our knowledge, no studies have described any particular needs that CI users with SSD may have regarding mapping strategies. One of the challenges for the clinician providing auditory rehabilitation for CI users with SSD is to ensure that the sound processor is optimally fit. Although improving speech perception in noise is the ultimate goal, speech perception scores themselves do not provide the clinician with specific information that would guide programming of the sound processor. Measuring the cortical auditory evoked potentials (CAEPs) in response to sounds that span the speech frequency spectrum may be a valuable tool for clinicians to verify detection and audibility of speech sounds in CI users with SSD and thereby, optimize fitting.

Several studies have investigated the use of CAEPs to verify speech detection based on evoked neural activity at the cortical level in children and in adults receiving amplification [21–28] and research has demonstrated a clear relationship between speech perception and the presence of CAEPs [29].

In adults, CAEPs consist of a positive peak (P1) at approximately 50 msec, a negative peak (N1) around 100 msec, followed by another positive peak (P2) around 180 msec [30]. The presence of this complex P1-N1-P2 indicates that that auditory cortex has been activated by the sound and when a CAEP response is recorded in response to speech signal it may be valuable evidence that the speech is audible to the subject [26,31–33]. With this in mind, the aim of this study was investigate the use of CAEPs to objectively evaluate the fittings of CI users with SSD.

## Materials and methods

### Subjects

To be included in the study, potential subjects had to:

- have postlingual SSD, defined as having mean pure tone average of ≥70 dB HL in the ipsilateral ear and ≤ 30 dB HL in the contralateral ear (at 0.5, 1, 2, and 4 kHz),
- have been using the CI speech processor on a full-time basis for 3–36 months.

This study was designed and conducted in accordance with the Helsinki Declaration. Ethical and institutional review committee approvals were obtained from the South Metropolitan Area Health Service Ethics Committee. Written informed consent was obtained from all subjects.

### Testing setup

Sound field cortical responses to the speech tokens /m/, /g/, /t/, and /s/ were recorded for each subject. These speech tokens were chosen because they span the frequency range important for language. The stimuli were presented at the soft presentation level of 55 dB SPL using the HEARLab System (Frye Electronics, Tigard, Oregon) software version 1.1.1312.01. This level allowed the masking level in the contralateral ear to be lower and therefore more confortable for the participant. The speech signal duration was 79ms; the inter stimulus interval was 1125ms; and the minimum number of acceptable epochs for responses to each speech signal was set at 200 [34].

The HEARLab is a commercially available single-channel recording system which is used to evaluate hearing aid fittings in infants and children. HEARLab applies an automatic statistical criterion to determine the presence or absence of CAEPs [28]. The automatic statistical criterion have been detailed by Golding et al. [35]. During the test, the ongoing statistical analysis results are displayed in the Detection p plots. The detection p-value indicates the probability that the response is significantly different than noise with a p-value < 0.05 indicating a significant result. The averaged responses to all stimuli are shown in the Cumulative Average plot for visual inspection of P1-N1-P2 complex waveform. Carter et al. [36] demonstrated in their study that the automated statistical detection of cortical responses used in the HEARLab system is as accurate as visual detection by 3 expert CAEPs examiners.

Testing was performed in free-field in a soundproofed room with subjects seated 1 meter away from a loudspeaker located at ear level at 0°. The contralateral (normal hearing) ear was masked with 70 dB HL broad-band noise presented through an insert earphone.

All subjects were tested using their routine map, which was created as per standard clinical care of a CI recipient. Subjects sat still and remained alert during testing either by reading a book or watching a movie without sound. The electrode montage was as follows: active on the vertex, reference on the contralateral mastoid, and ground on the forehead. Impedance was kept below 5 k Ohms. The time window for the recording was from -200ms (baseline) to 600ms. The residual noise level was kept below 3.2 μV. The number of accepted epochs in a test run was preset to 200 epochs.

### Calibration to determine sufficient masking

**Fig 1:**
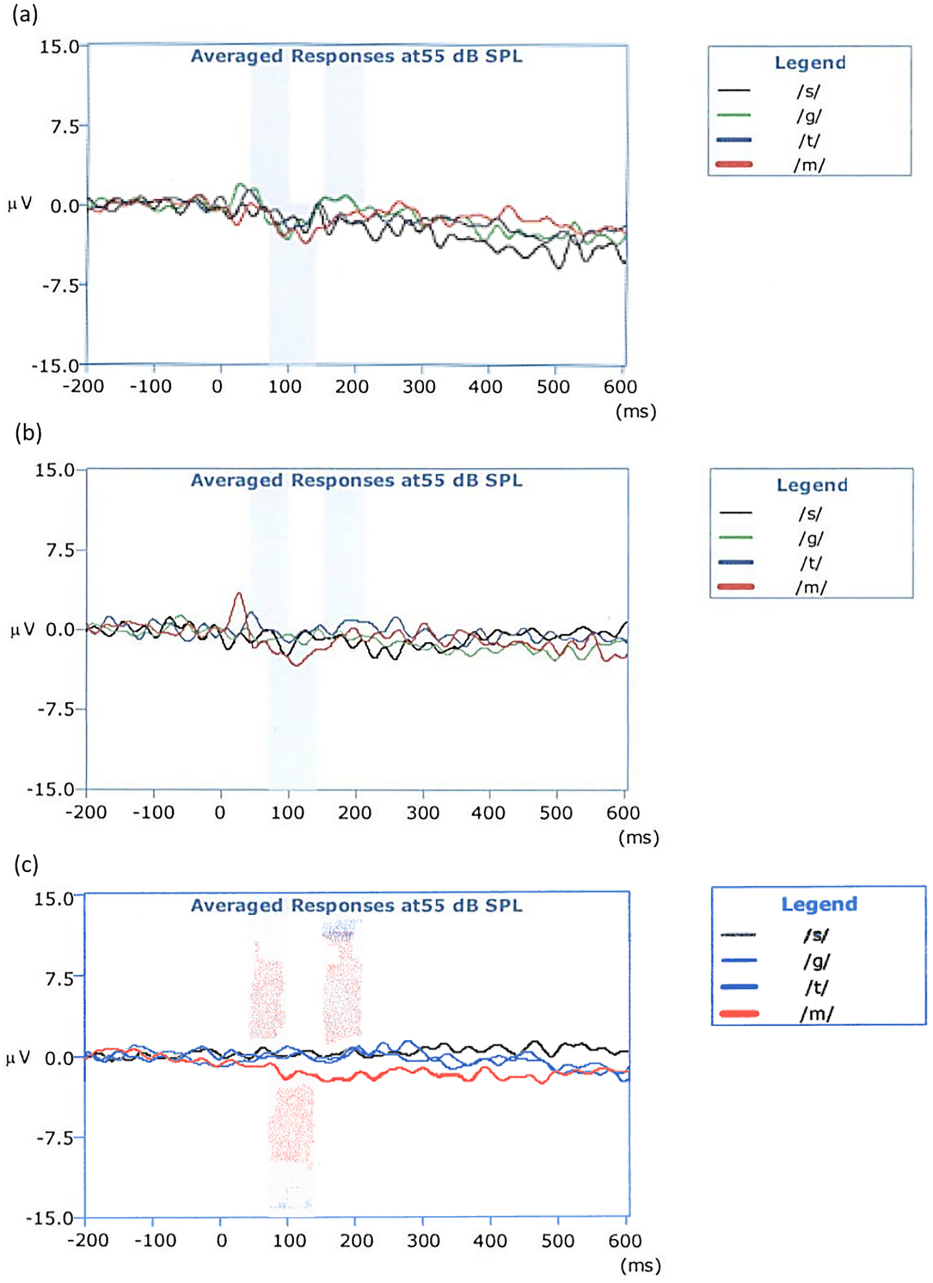
Subject S1’s CAEP traces evoked by speech tokens /s/, /g/, /t/, /m/ with CI off and his normal hearing ear masked with 60 dB HL (1a), 65 dB HL (1b), and 70 dB HL (1c) broad-band noise.

Sufficient masking was obtained at 70 dB for the first three subjects, therefore 70 dB HL was used for all remaining testing. To ensure that 70 dB HL provided sufficient masking, all subjects were tested in the CI-off condition to check for the absence of a CAEP waveform. See Fig 1 for an example.

### Aided CAEPs

CAEP responses were also evaluated by looking at the presence in the waveform of the peaks P1, N1, P2 [37]. An audiologist who was blinded to the programming of the implant and to the HEARLab results performed this task. A clinical implant audiologist programmed the implant, and a third audiologist who was blinded to the review of the first audiologist reviewed the waveform to ensure that reliable peaks were identified. If the HEARLab software indicated a present cortical response (p-value <0.05) and the waveform was considered to be cortical a response as per at least one audiologist, the subject’s fitting was regarded as optimal. If, however, no responses were detected by the HEARLab and at least one audiologist was unsure about the waveform, a new map was created by gradually increasing the most comfortable levels (MCL) for the electrode contacts corresponding to the speech tokens that did not evoke a CAEP. Subjects were instructed to alert the audiologist if the new levels became uncomfortable or provoked non-auditory stimulation. CAEP responses were measured after each adjustment and further adjustments were made until a CAEP was evoked. After creation of the fitting, subjects were asked to use their new setting for 2–3 weeks for acclimatization purposes and then return for retesting. At retesting, new CAEP recordings were performed to objectively ensure that the new fitting maps effectively activated the auditory cortex.

## Results

### Subjects

Nineteen CI users (10 male, 9 female) met the inclusion criteria and participated in the study. Subjects’ mean age at implantation was 50.8 years (range 23–74 years) and mean duration of CI experience was 9.1 years (range 3 months– 39 years). The most common etiology (12 subjects) was idiopathic sudden sensorineural hearing loss. All subjects used a STANDARD or FLEX28 electrode array and an OPUS 2, RONDO, or SONNET speech processor (MED-EL, Innsbruck, Austria) (See Table 1).

**Table 1:** Demographic data.

ID	Years old at CI implantation	Duration of deafness (years)	Sex	Ear	Etiology	Pure tone average (dB)	
						Non implanted ear	Implanted ear
S1	56	2	M	L	Head trauma	21	100
S2	41	12	M	L	ISSNHL	5	76
S3	71	20	M	R	ISSNHL	25	83
S4	48	0.9	F	L	ISSNHL	7	75
S5	43	0.6	M	L	ISSNHL	14	93
S6	55	1	M	L	Meniere’s	25	75
S7	42	0.2	F	R	Meningitis	30	75
S8	70	3	F	L	ISSNHL	11	78
S9	52	0.4	M	R	ISSNHL	14	79
S10	34	0.8	F	R	ISSNHL	10	90
S11	42	1	M	R	ISSNHL	5	90
S12	49	30	M	L	ISSNHL	10	>110
S13	70	0.8	M	R	ISSNHL	7	70
S14	46	40	F	L	Mumps	11	>110
S15	59	0.6	F	L	Meniere’s	20	75
S16	55	40	F	L	ISSNHL	12	80
S17	36	0.3	F	R	Fistula	27	>110
S18	74	1.4	M	R	Ear Surgery	22	77
S19	23	17	F	R	ISSNHL	15	>110

ISSNHL: Idiopathic Sudden Sensorineural Hearing Loss Syndrome.

### CAEP measurements

For 14/19 subjects, CAEPs were detected for each speech token as pear HEARLab and at least one audiologist. These 14 subjects did not have their maps adjusted. In the other 5 subjects (#s S9, S10, S12, S15, S17) CAEPs could not be evoked by at least one speech token. In all 5 cases, at least one audiologist was unsure if there was a cortical response as per visual inspection. These 5 subjects had their fitting maps adjusted as follows:

- Subject S9: no clear CAEPs were evoked by any of the speech tokens. This was consistent with his perception that the sound processor was softer than usual. After fitting him with a new sound processor, CAEPs were detected for all 4 speech tokens.
- Subject S10: no clear CAEPs were evoked by /s/. CAEPs were detected after a two-step increase in the high frequency MCLs.
- Subject S12: no clear CAEPs were evoked by /g/ or /m/. CAEPs were detected after low and mid frequency electrodes were adjusted once.
- Subject S15: no clear CAEPs were evoked by /t/. CAEPs were detected after a one step increase in mid-high frequencies electrode contacts.
- Subject S17: no clear CAEPs were evoked by /s/ or /t/. After the first MCLs increase in the mid-high frequency electrode contacts, CAEP responses could be recorded for /t/ but not for /s/. The immediate increase in MCLs was reported by the subject as “too high pitched”. Despite this, he was happy to try to acclimatize to the new setting and use the new map at home. He was therefore asked to use the sound processor for 3 weeks and return for a review. At the review he reported that he had adjusted to the map. CAEPs could be recorded for all 4 speech tokens.

**Fig 2:**
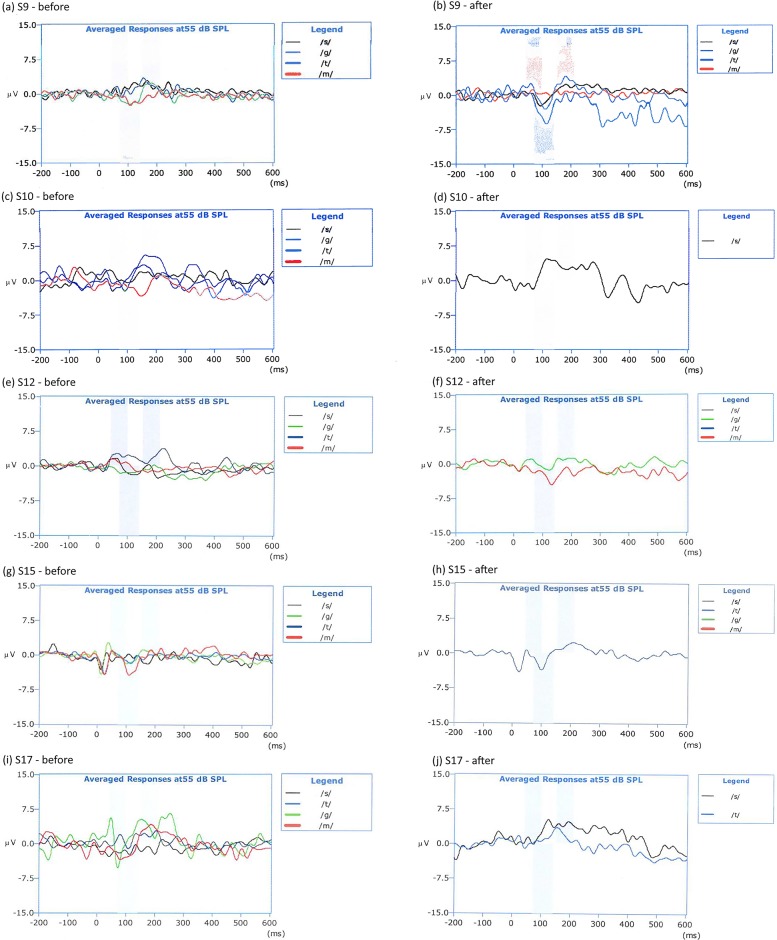
Before and after CAEP traces for the 5 subjects (#s 9, 10, 12, 15, 17) whose fitting maps had to be adjusted.

All subjects adapted well to their new map and none have asked to return to their previous setting. All subjects reported that the new settings resulted in an improved overall sound clarity. (Fig 2).

## Discussion

The aim of the present study—to use CAEPs to optimize CI fitting in experienced adult CI users with SSD—was accomplished. While 14/19 subjects did not need any adjustments to their CI settings, the other 5/19 subjects had their maps adjusted to enable and enhance full access to speech sounds. When these 5 subjects were reviewed 2–4 weeks post-adjustment, all had adapted well to their new mapping settings and none wished to return to their previous fitting. These short-term results are nonetheless encouraging.

For people with SSD, CI is the only hearing rehabilitation option that provides ear-specific information and therefore the potential of binaural hearing benefits. The two main goals of hearing rehabilitation with a CI are 1) better speech understanding and 2) an improved ability to localize sound sources. It is reasonable to expect that hearing across the speech spectrum via a CI would facilitate a balanced hearing input from both ears and therefore enhance binaural hearing benefits.

Although most adults are perfectly capable of providing feedback during CI fitting sessions, in our experience it is common to see that some CI users tend to be quite conservative on how they define MCL whilst others are more tolerant of increased MCL. This subjectivity method could result in electrical stimulation being either insufficient or unpleasantly loud, both of which would likely diminish the compliance rate and impede the rehabilitation process. Subjective bilateral loudness balancing, i.e. asking the CI user to match the loudness from the CI side to the normal hearing ear, can often be used with adults but is impossible or impractical with some children.

There is already growing interest in using CAEPs to objectively determine if pediatric hearing aid users can detect speech sounds at conversation levels [24,38,39], similarly, this present study evaluated if CAEPs could be used to objectively measure if adult CI users with SSD can detect speech sounds at conversational levels in the CI-alone condition. This technique if used with recently implanted subjects could potentially indicate that the CI settings are enabling the patient to have access to the speech spectrum at an early post-implantation stage.

In the authors’ clinical experience, optimal fittings are essential at this early stage because CI users are more committed to auditory training if they derive early benefit from CI use. Consequently, using CAEPs to verify the suitability of fitting may improve the overall outcome *and* reduce the number of follow-up appointments.

It is important to consider that access to speech sound does not necessarily imply good speech understanding because other factors may be involved. However, access to speech sounds is the first step to improving speech understanding. It would be interesting to investigate if speech understanding scores correlate with CAEPs in patients fitted through this method and also compare the outcomes of patients fitted using the traditional subjective method to those fitted using CAEPs. In addition, a comparison of the ipsi- and contralateral auditory pathways in CI users with SSD would expand our understanding of this group’s specific needs; this is currently under investigation as a follow up of the present study.

## Conclusion

Using CAEPs is a quick and objective method to evaluate the fittings adult CI users with SSD at an early post-implantation stage. Such evaluation and, if necessary, correction, could expedite their hearing rehabilitation process.
